# Detail in Cleft Palate Operations.—Abstract

**Published:** 1899-11

**Authors:** Stewart L. M’Curdy

**Affiliations:** Pittsburg, Pa.


### Detail in Cleft Palate Operations.—Abstract.

BY STEWART L. M'CURDY, M.D., PITTSBURG, PA.

Presented to the Section of Surgery and Anatomy, at the Fiftieth Annual Meeting of the American Medical Association, held at Columbus, Ohio, June 6-9, 1899.

The object of this paper is to describe some new instruments and to call attention to some already in use, as well as to describe the accepted methods of operation for congenital defects of the hard and soft palate.

In all operations on the mouth or face the patient should be placed in the Murphy position, the operator sitting above the head. The Murphy position is best secured by the use of the head-rest of an ordinary operating table, or by an extra head-rest with a body portion passed under the patient—the head-piece being placed at an angle of forty-five degrees to the plane of the body.

Fillebrown's method of producing anesthesia is of advantage in all operations on the face and mouth. It is practically the same method as that introduced by Dr. Hayes, a dentist of Pittsburg, about fifteen years ago. It consists in delivering the anesthetic to the point through a small tube near the mouth, or as Fillebrown suggests, resting on the upper teeth. The anesthetic is forced through the tube by a foot bellows. With the patient in position, the Fillebrown mouth-piece can not be used as he suggests. When possible the Davies Colley operation is to be preferred, since it furnishes normal mucous surface for the floor of the nasal cavity and for the roof of the mouth as well, thus avoiding the exposure of raw surface. He describes the steps of the operation as follows:

The operation might be divided into three stages. Stage 1. An incision is made down to the hard palate in front, and behind through the soft palate, with its center just internal to the last molar. With a raspatory the muco-periosteum is separated from inward. An incision is made about a quarter of an inch from the cleft in front. It runs parallel to the cleft backward, and is continued to the tip of the uvula, splitting the soft palate to the depth of about three-eighths of an inch in front and a less amount behind. The muco-periosteum and the cleft of the hard plate is separated inward with the raspatory as far as the edge of the bone. A triangular flap is raised from the other side of the plate in such a way that the anterior extremity is free, and the inner margin runs parallel to the edge of the cleft at a distance of one-sixth of an inch. In the soft plate the incision is continued backward so as to split that structure in the same way as on the other side. The muco-periosteum of the hard palate internal is separated inward and left attached to the edge of the bone.

Stage 2. The mesial flaps are united by fine silk or catgut sutures; and continuously with this union the upper planes of the split soft palate are brought together. A bridge is thus formed across the whole cleft with a mucous surface directed upward and a raw surface downward.

Stage 3. The edge of the triangular flap in front, and of the lower plane of the soft palate behind, is united by silver wires and one or two silk sutures, to the edge of the hard and soft palate of the other side. A second bridge is thus formed across the whole cleft, with a raw surface looking upward and a mucous surface downward. The after-treatment was that of the ordinary operation, except that as there was no tension in the hard palate, and very little in the soft, the sutures might be left in from three to six weeks. The advantages claimed for this operation were: 1. No tissue has to be pared away; 2, a much larger extent of raw surface is brought into closer contact than in the ordinary operation; 3, the tension is small; 4, the upward pressure of the tongue is beneficial, as it presses the lower er against the upper bridge; 5, some advantage is gained by using the muco-periosteum of one side in front of the cleft to help in bridging the gap of the hard palate. After paring the edges of the flaps I inserted silver sutures, running above the flaps, and passed them through the palate and through pure silver disks, twisting the ends and making tension which fully approximated the edges. To fully relieve the strain upon the soft palate, and also to allow contraction of the arch, I made the incision from the tuberosity to opposite the under molar. I cut through the mucous membrane with a scalpel and then separated the deeper tissues with a smooth dissector, in order to avoid injuring important vessels. The edges of the cleft, now held in close approximation, were brought more accurately together by fine silk sutures. I thus avoided the lateral incision in the hard palate, the separating of the tensor muscles, the cutting of the pillars of the fauces, and approximated the tonsils so as to lessen the arch and allow the soft palate to retract to a considerable extent. This increases the length of the flap. If the flap is short near the fauces an incision is made through the mucous membrane near the anterior pillars.

Deep wire sutures and head plates are used by the operator to relieve tension on the flaps as well as marginal sutures.

Dr. Truman W. Brophy, Chicago, recommends that the maxillary bones be approximated by twisting silver wire which has been passed through the aveolar ridges.

In every operation on congenital deformities the preservation of material must be the first consideration. The least amount of tissue must not be severed. In the Davies Colley operation this is carried out throughout. In Fillebrown's operation a skillful step toward economy of tissue is observed. The instrument used is a steel hoe with cutting edge on the tip and both sides. It was devised for the purpose of cutting through the soft tissues well up around the margin of the cleft, indeed, far enough to so do that when the flaps are hoed from the bone they reach across the chasm. With the cutting sides it is drawn forward to the anterior angle and backward along the margin of the soft palate, as well as hard—the uvula being very readily split to its tip. With care a considerable cleft may be closed in this manner without resorting to bone operation, as Brophy suggests.

To use a needle without an ordinary needle-holder requires a very small needle and skillful manipulation, and much time is consumed. The cervix needles, which have the eye near the point, and are bent with a curve at right angles to the staff, answer quite well through the uvula, but are most difficult to handle between the bony cleft. The usual staphylorraphy needle is bent with a slight curve at right angles to the staff, with a very slight curve of the point back toward the handle.

To overcome this difficulty the needle herewith illustrated was devised. It has a curve which brings the point back toward the handle, the curve being just great enough to carry the puncture back sufficiently far from the edge of the flap. The instrument used here is necessary to complete the introduction of the suture skillfully. It has a ring on one end, bent at right angles to the staff, and with an opening large enough to admit the threaded needle. On the other end is a hook. The needle, after being loaded with the desired suture material, is passed through back of the flap into the nasal cavity. It is then pushed through the flap from the nasal into the oral cavity. The ring end of the instrument containing the hook and ring above described is pressed against the flap to support it and prevent laceration. After the needle and suture passes through the flap, the hook is made to engage the suture on the side of the needle from the staff, and with traction the free end is drawn through into the oral cavity as the needle is pushed back into the nasal cavity. The needle, with the thread still in position, is turned around to the opposite side, and passed through the flap as above described. As the thread is hooked up on the inside of the needle, the salient end on the same side is let go, and the one on the opposite side held—otherwise the needle would be unthreaded. The hook is again passed around the suture on the outside of the needle as before, and as it is drawn through into the oral cavity, the needle is pushed back into the nasal cavity and drawn out between the flaps unthreaded. This method permits the introduction of sutures at the extreme anterior angle. It is done quickly and no undue violence is done to the flaps. An important detail is that the hook must be passed around the suture on the side of the needle next the staff, on both sides, or when it is withdrawn, instead of it unthreading itself, it remains threaded and the two ends are through the flaps on the two sides of the cleft. The thread may be drawn from the inside of the needle, on both sides, and the steps are just the same.

A self-retaining mouth gag adds greatly to the convenience, and greatly assists in expediting the operation. For this purpose, the instrument herewith was devised. It is made as an ordinary gag, except that it has a plate extending from the end of the upper arm of the gag, about the side of the head, above and around the ear. The plate which passes about the side of the head is made of malleable metal, so that it can be accurately adjusted to the side of the head without tilting the mouth gag from the teeth. This is held in position by a head-band, which may be an ordinary sterilized gauze bandage. With this simple device the assistant is free to render double service. A self-retaining tongue depressor is attached to the gag. The tongue can be thrown up to any desired position by moving the lever, which is pivoted on the lower arm of the gag. The outer lever of the tongue plate is bent along the lower bar of the gag to near the end, where it is turned up at right angles, this part passing through an opening on the lower arm of the gag. It is secured by a ratchet or thumb screw, as may be desired. As the gag is closed, the pressure made by the tongue plate is relieved, and as it is opened the tongue is thrown up out of the field of operation.

